# Valorization of Unused, Expired Surgical Masks in Polymer-Modified Bitumens Using Maleic Anhydride as a Compatibilizer

**DOI:** 10.3390/polym17233110

**Published:** 2025-11-23

**Authors:** Paola Scarfato, Sabino De Gisi, Annalisa Apicella, Marinella Levi, Nadka Tz. Dintcheva, Loredana Incarnato

**Affiliations:** 1 Department of Industrial Engineering, University of Salerno, Via Giovanni Paolo II 132, 84084 Fisciano, SA, Italy; pscarfato@unisa.it (P.S.); anapicella@unisa.it (A.A.); lincarnato@unisa.it (L.I.); 2 Department of Chemistry, Materials and Chemical Engineering, Politecnico di Milano, Piazza Leonardo da Vinci 32, 20133 Milano, MI, Italy; marinella.levi@polimi.it; 3 Department of Engineering, University of Palermo, Viale delle Scienze, Ed. 6, 90128 Palermo, PA, Italy; nadka.dintcheva@unipa.it

**Keywords:** infrared spectroscopy, maleic anhydride, materialistic characterization, material substitution, penetration force, softening measurements, viscosity

## Abstract

In this study, polypropylene (PP) recovered from unused, expired surgical masks was evaluated as a substitute for virgin PP in polymer-modified bitumen (PMB). Unlike previous studies that incorporated whole masks or mixed polymer residues into bitumen, this work focuses specifically on recovering and functionalizing the polypropylene layers of surgical masks to directly replace virgin PP in PMB formulations. To improve the compatibility between PP and the bituminous matrix, maleic anhydride (MAH) and a maleic anhydride-grafted compatibilizer (AUS) were incorporated through different blending strategies. Five PMB formulations (0.5–5 wt.% polymer content) were prepared from B_70/100_ reference bitumen. ATR/FT-IR confirmed the absence of thermo-oxidative degradation during mixing. Viscosity, penetration force and softening behaviour tests at 10–40 °C identified the MAH-functionalized mask-derived PP (PMB_MMAH) as the best-performing formulation. Compared to the base bitumen, this formulation increased the softening point by ~10–15 °C, raised viscosity by ~20–30%, and reduced penetration by up to 25%. These results demonstrate that mask-derived PP can provide a sustainable alternative to virgin PP while ensuring comparable or improved technical performance. Further studies will evaluate long-term ageing behaviour and environmental impact.

## 1. Introduction

During the COVID-19 pandemic, the increased use of face masks as a preventive measure against the virus resulted in significant waste generation, particularly from disposable masks [1]. Moreover, compulsive purchases by governments during the pandemic led to the accumulation of large quantities of undistributed and unused masks. An estimated 129 billion masks per month have been consumed worldwide since the start of the pandemic, with over 1.24 trillion masks discarded globally between December 2019 and May 2021 [2,3]. Despite having economic value as products, these masks are classified as waste and, at best, are destined for energy recovery or, alternatively, for landfill disposal or direct release into the environment [4,5]. In all cases, these waste management strategies have significant environmental impacts, including the production of greenhouse gas emissions [6,7].

In recent years, various strategies have been explored for the recovery and recycling of plastic waste from surgical masks. Lyu et al. [8] provided an overview of recently developed methods for the valorization of plastic waste from face masks, which are categorized into mechanical reprocessing [9,10,11], thermochemical recycling [12,13], pyrolysis upcycling and chemical reuse [14,15,16]. Research on mask recycling has pursued multiple objectives, such as recovering valuable chemicals and materials (e.g., hydrogen or additives for cement mortars and bitumen), exploring their energy valorization, and conducting Life Cycle Assessment (LCA)-based evaluations [17,18]. Another promising approach involves incorporating mask-derived polymers into bitumen, as conventional bitumen is prone to temperature-related performance losses and ageing, and therefore commonly requires polymeric modifiers to improve its mechanical stability and long-term durability.

Polymer-Modified Bitumen (PMB) refers to bituminous binders in which specific polymers are added to enhance elasticity, resistance to deformation, and service performance under varying climatic and traffic conditions. The most commonly used polymers for bitumen modification are elastomers—such as styrene–butadiene–styrene (SBS), styrene–isoprene–styrene (SIS), and styrene–ethylene/butylene–styrene (SEBS) [19]—and plastomers, including polyethylene (PE), polypropylene (PP), ethylene–vinyl acetate (EVA), and ethylene-butyl acrylate (EBA). Elastomeric polymers improve the elasticity, flexibility, low-temperature resistance, rutting resistance, ageing resistance, aggregate adhesion, frost cycle resistance, and performance of bitumen in extreme weather conditions. Plastomeric polymers, on the other hand, enhance thermal resistance to high temperatures, rigidity, abrasion resistance, and durability in aggressive environments. These distinct properties determine the different application areas for each type of polymer.

Although significant progress has been made in the field of PMB, several limitations still hinder its widespread use, including the storage stability of certain PMB formulations and the incompatibility between the bitumen matrix and the polymer modifier [20,21,22,23,24]. PP and PE exhibit poor compatibility with bitumen, whereas SBS and EVA provide more satisfactory results.

To address these limitations, various techniques have been developed, including saturation, vulcanization, antioxidants, hydrophobic mineral clays, functionalization, and radical grafting compatibilization [25,26]. In the context of functionalization and radical grafting compatibilization, maleic anhydride (MAH) is an unsaturated cyclic compound (five-carbon-atom ring, molecular formula C_4_H_2_O_3_) used as a compatibilizer between bitumen and polymer. In the molten state, MAH undergoes a radical grafting process, covalently bonding to PP, which increases its polarity [27,28]. This improved polarity enhances its dispersion within the bitumen, leading to greater stability and improved overall performance of the compound [29]. However, PMB production remains relatively expensive, mainly because it requires virgin polymers and additional polymer treatments (e.g., vulcanization). Consequently, increasing attention has been directed towards recycled materials, particularly PP and PE, which are abundantly available in municipal solid waste [30]. Research in this area has explored several approaches, including (i) utilizing cross-linkable recycled polyethylene (PE) with silane derived from waste [31], (ii) examining the rheological, chemical, and morphological changes in high-content PMB under long-term oxidative thermal ageing [32], (iii) substituting virgin polymers with recycled linear low-density polyethylene (RLLDPE) in PMB production [33], and (iv) incorporating waste masks into PMBs using styrene-butadiene rubber-styrene (SBRS) as a compatibilizer and shredding the masks to include all their components [34]. Despite these advancements, limited research has focused on surgical masks—or their components—as a replacement for virgin polymers in PMB applications. Additionally, commercial PMB products often lack transparency regarding the specific polymers and additives used, highlighting the need for further investigation.

Therefore, this study aims to evaluate the technical feasibility of recycling PP components from unused surgical masks into reference bitumen as a substitute for virgin PP. To improve compatibility between the polymer and bitumen, compatibilizers—specifically maleic anhydride (MAH) and AUS (a maleic anhydride-grafted compound)—were incorporated into the mixtures using different application methods, as described in this study.

## 2. Materials and Methods

### 2.1. Experimental Plan

The main phases of the experimentation included the characterization of the materials used in the surgical masks, the preparation of the solid phase for incorporation into the bituminous mixture, the preparation of the bituminous mixtures themselves, their characterization, and the comparison of the resulting performance values with those of the reference bitumen. 

### 2.2. Materials

**Figure 1:**
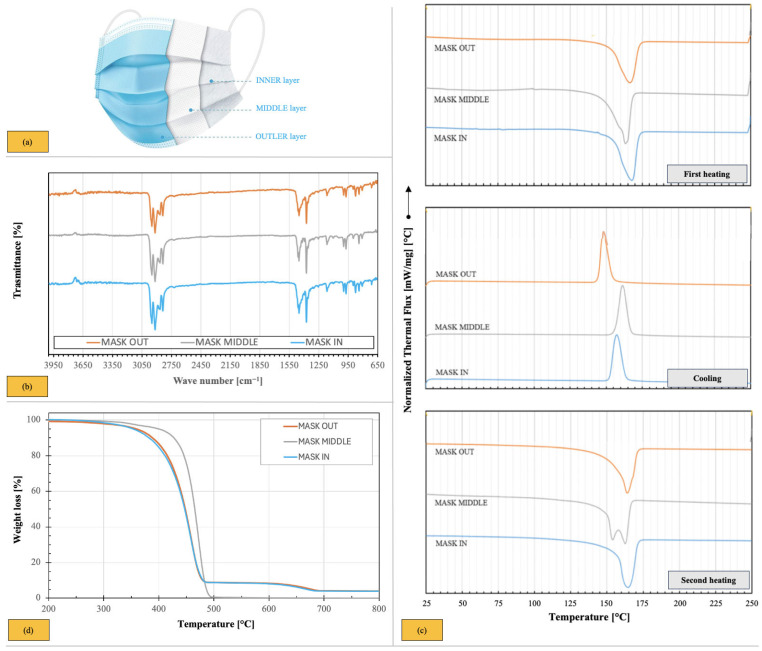
Characterization of the masks under study: (**a**) schematization of the three-layer non-woven surgical mask to which the analytical determinations below refer; (**b**) ATR/FT-IR analyses; (**c**) DSC thermograms; (**d**) thermogravimetric spectrum.

The surgical masks used in this study were unused products that had reached the end of their certified shelf-life. As they had never been worn or exposed to medical or public environments, they did not present any biohazard risk. Masks from five manufacturers were selected to ensure representative variability. The total weight of a single face mask was 3.24 ± 0.01 g, consisting of 78.6% filtering face mask, 12.6% ear loop, and 8.8% nose wire. Only the three filtering layers (outer layer, middle layer, and inner layer, see Figure 1a), all made of PP nonwoven fabric, were used as modifiers for the bitumen after being shredded into 3–5 mm fragments.

Virgin PP (Moplen RP241H, melt flow rate (230 °C/2.16 kg): 1.8 g/10 min), supplied by LyondellBasell (Milan, Italy), was used as a reference material for comparison.

As a compatibilizer, a compound consisting of a PE/PP blend derived from mixed polyolefin packaging waste and grafted with MAH was considered. This compound, supplied by Auserpolimeri (Lucca, Italy), is hereafter referred to as AUS (Tg = −58 °C, Tm = 67.8 °C). The maleic anhydride content in AUS was approximately 0.5 wt.% [35].

As binders, three paving-grade bitumens with penetration grades of 35/50, 50/75 and 70/100, supplied by Eni SpA (Rome, Italy), were used. The general properties of these bitumens are reported in Table 1. The 70/100 bitumen was used as the reference for comparison with the obtained results.

All the reagents used for the functionalization experiments and titration analyses–including MAH, dicumyl peroxide (DCP), potassium hydroxide (KOH), hydrochloric acid (HCl), acetone, and xylene-were reagent-grade, supplied by Sigma Aldrich (Milan, Italy), and used without further purification.

The first step in processing the masks involved disassembling their components. Subsequently, each of the three parts underwent the following characterizations: (i) ATR/FT-IR analysis, (ii) DSC, (iii) TGA, (iv) density testing, and (v) mechanical tensile testing. The methods and equipment used are described in Section 2.4.

### 2.3. Modified Bitumen Preparation

PMBs were prepared using the following PP modifiers: (i) shredded masks; (ii) shredded masks with the addition of 5 wt.% AUS compatibilizer; (iii) shredded masks impregnated with an acetone solution containing 0.8 wt.% MAH as a functionalizing agent and 0.1 wt.% dicumyl-peroxide as a radical initiator, then left to dry under a fume hood before proceeding; and (iv) virgin PP, used for comparison purposes.

The PP modifiers were gradually added at concentrations of 0.5 wt.%, 1 wt.%, 3 wt.%, and 5 wt.% to the pure bitumens, which had been preheated to 180 °C until fully liquefied. The mixtures were then stirred at this temperature at 1000 rpm for 30 min. Under these conditions, the MAH impregnating the shredded masks in PP modifier (iii) grafts onto the PP through a radical mechanism, leading to its functionalization [39].

To assess the effects of the PMB preparation process on bitumen performance, the pure bitumens were also subjected to the same mixing procedure.

The nomenclature and composition of the PMB mixtures are summarized in Table 2.

Three samples were prepared for each PMB mixture, and the mean and standard deviation were calculated for each characterization parameter.

### 2.4. Modified Bitumen Characterization

#### 2.4.1. Subsubsection

Fourier transform infrared spectroscopy analysis in attenuated total reflectance mode (ATR/FT-IR) was performed using a ThermoNicolet NEXUS 600 spectrophotometer (Thermo Fischer Scientific, Waltham, MA, USA), equipped with a SmartPerformer accessory. Each spectrum was recorded in the 4000 ÷ 600 cm^−1^ frequency range, with a resolution of 4 cm^−1^ and 64 co-added scans.

#### 2.4.2. Differential Scanning Calorimetry (DSC)

DSC analyses were conducted using a DSC Model 822 apparatus (Mettler-Toledo International Inc., Columbus, OH, USA) under a nitrogen gas flow (100 mL/min) to minimize thermo-oxidative degradation phenomena. The following temperature programme was applied: heating from 25 °C to 200 °C at 10 °C/min, holding at 200 °C for 5 min, cooling to 25 °C at 10 °C/min, and reheating to 200 °C at 10 °C/min. The percent crystallinity was determined using the following equation [Equation (1)]:

% Crystallinity = [∆Hm − ∆Hc]/∆Hm^0^ × 100% (1)

where ∆Hm^0^ = 170 J/g is a reference value representing the heat of melting for 100% crystalline PP [40].

#### 2.4.3. Thermogravimetric Analysis (TGA)

TGA was performed using a TA-50 WSI thermal analyzer (Shimadzu Italia S.r.l., Milan, Italy). Samples (10–14 mg) were heated from 25 °C to 800 °C at 10 °C/min under a nitrogen atmosphere (flow rate: 50 mL/min).

The purpose of the TGA was not to reproduce the processing conditions, but to assess the thermal stability of the mask-derived PP and to determine the inorganic residue (e.g., CaCO_3_). The upper temperature limit, therefore, allows the identification of the onset of polymer degradation and provides a safety margin relative to the PMB mixing temperature (<180 °C).

#### 2.4.4. Density

A balance equipped with a device for measuring the weight of a solid in air and when immersed in a liquid of known density was used. The technique is based on Archimedes’ principle, which states that a body partially or fully immersed in a fluid experiences an upward buoyant force equal to the weight of the fluid it displaces. The material under test was first weighed in air and then in ethanol (a liquid with a known density), from which its density was determined using standard mathematical relationships.

#### 2.4.5. Viscosity of Bituminous Mixtures

The viscosity of both pure bitumens and PMBs was measured using a rotational viscometer (Thermo Scientific HAAKE Viscotester C, Fisher Scientific Italia, Segrate, Italy) in accordance with UNI EN ISO 2555 [41]. Tests were performed in triplicate at 135 °C on specimens weighing approximately 300 g.

#### 2.4.6. Penetration Tests

To evaluate bitumen consistency, penetration tests specifically designed for bituminous mixtures were conducted. A CMT4000 SANS dynamometer (MTS, Changzhou, China) was used, in which a cylindrical, pointed bamboo tool acted as a needle, penetrating the bituminous mixture by means of a crosshead moving at a constant speed of 120 mm/min. A 100 N load cell was employed. The test involved penetrating the mixture to a depth of 10 mm and recording the penetration force and penetration energy values. The bamboo tool measured 19.5 cm in length and 4 mm in diameter, tapering to a point over the last 2.4 cm. Tests were carried out at four different temperatures: 10 °C, 20 °C, 30 °C, and 40 °C.

#### 2.4.7. Softening Tests

Softening tests were conducted to compare the softening behaviour of the investigated PMBs with that of the corresponding pure bitumen, which served as the reference. For each PMB, three samples with a diameter of 4 cm and a height of 0.5 cm (approx. 8 g) were prepared and placed at the centre of a plate holder featuring concentric circles. The innermost circle had a diameter of 4 cm, the outermost 10 cm, with 2 mm spacing between each circle (Figure S1, Supplementary Material). The samples were maintained at three different temperatures (4 °C, 20 °C, and 30 °C), and their diameter expansion was measured at various softening times, up to a maximum of 48 h. The average results were then plotted, as shown in Figure S1 (see Supplementary Material).

#### 2.4.8. Determination of MAH Grafting Degree

The MAH grafting degree was determined using a back-titration method.

Approximately 1 g of functionalized shredded masks was dissolved in 100 mL of hot xylene (approx. 190 °C). The hot solution was immediately titrated with 0.01 N KOH in ethanol, using 3–4 drops of a 1% thymol blue indicator. About 1 mL of excess KOH solution was then added, and the intense blue colour was back-titrated to a yellow endpoint by adding a 0.1 N HCl solution in 2-propanol [42].

The amount of grafted MAH was calculated using the following equations [Equations (2) and (3)] [42]:

(2) $$A\mathrm{cid}\;\mathrm{No}\;\left(\frac{{\mathrm{mg}}_{\mathrm{KOH}}}{g_{\mathrm{masks}}}\right)=\frac{\left[{\mathrm{mL}}_{\mathrm{KOH}}+\left(1{\mathrm{mL}}_{\mathrm{excess}\;\mathrm{KOH}}-{\mathrm{mL}}_{\mathrm{HCl}}\right)\right]\times N_{\mathrm{KOH}}\times 56.1}{g_{\mathrm{masks}}}$$

(3) $$M\mathrm{AH}\;\%=\frac{\left(\mathrm{Acid}\;\mathrm{No}\times 98\right)}{\left(2\times 56.1\right)}$$

where *N* is the normality of the solution, 56.1 u.m.a. is the molecular weight of KOH, and 98 u.m.a. is the molecular weight of MAH.

### 2.5. PMBs Performance Comparison

The comparison considered both the individual key parameters of bituminous mixtures specified in UNI EN 14023 [43] (namely viscosity, penetration force, and softening point) and a composite performance indicator. While each key parameter provided insight into a specific aspect of the mixture, the composite indicator offered an overall assessment based on the combined evaluation of these parameters.

Denoted as CI, the composite indicator was constructed through several stages: (i) definition of the evaluation criteria, (ii) determination of the weights assigned to each criterion, (iii) elaboration of the alternative matrix, (iv) normalization of the alternative matrix, and (v) calculation of the CI index [44]. The alternatives matrix contained as many rows as the number of PMB mixtures and as many columns as there were evaluation criteria (in this case, three). Normalization of the matrix was performed using the max/min method, as described in Equations (4) and (5):

(4) $${\bar{x}}_{\mathrm{ij}}\;=\;\frac{x_{\mathrm{ij}}}{\mathrm{Max}\left(x_{j}\right)}$$

(5) $${\bar{x}}_{\mathrm{ij}}\;=\;\frac{\mathrm{Min}\left(x_{j}\right)}{x_{\mathrm{ij}}}$$

where x_ij_ represents the performance of the i-th alternative (each PMB mix, in terms of average value) concerning the j-th indicator (each evaluation criterion), w_j_ is the weight assigned to each indicator, and ${\bar{x}}_{\mathrm{ij}}$ is the normalized value of x_ij_, calculated using Equations (4) and (5) depending on whether the criterion was to be maximized or minimized. As a result, all values in the normalized matrix fell within the range [0, 1].

The CI index of each PMB mix was then calculated using Equation (6):

(6) $${\mathrm{CI}}_{i}=\overset{m}{\underset{j=1}{\sum }}{\bar{x}}_{\mathrm{ij}}\;\times {\;w}_{j}$$

The higher the CI value, the better the overall performance of the PMB mix, including when compared with the reference products.

For this calculation, all evaluation criteria were assumed to have equal weight.

## 3. Results and Discussion

### 3.1. Disposable Surgical Masks Characterization

ATR/FT-IR analysis was performed to confirm the composition of the three mask layers, which exhibited the typical absorption bands of isotactic PP. All spectra displayed characteristic PP peaks: –CH_3_ stretching at 2949 cm^−1^ (asymmetrical) and 2866 cm^−1^ (symmetrical), and –CH_3_ bending at 1376 cm^−1^ (symmetrical), –CH_2_– stretching at 2836 cm^−1^ (symmetrical) and 2916 cm^−1^ (asymmetrical), and –CH_2_– bending at 1455 cm^−1^ (symmetrical). Additionally, –CH_3_ rocking and CH wagging were observed at 1166, 996 and 972 cm^−1^; CH_2_ rocking and C-CH_3_ stretching at 840 cm^−1^; and C–C chain symmetric stretching at 807 cm^−1^ [45].

Notably, the spectrum of the inner layer slightly differed from the others, showing a pronounced shoulder at 1434 cm^−1^ on the peak at approximately 1455 cm^−1^, along with two additional peaks at 876 cm^−1^ and 712 cm^−1^ (Figure 1b). These features were attributed to the presence of calcium carbonate (CaCO_3_), commonly used in mask manufacturing as a whitener, stabilizer, and flame retardant [46].

The thermal behaviour of the masks was investigated through DSC analysis performed separately on each layer, with results presented in Figure 1c. During the first heating scan, all layers exhibited a melting peak within the temperature range of 162–168 °C, with similar crystallinity values between 37% and 42%. During the cooling scan, a single crystallization peak appeared in the range of 161–147 °C. Both melting and crystallization temperatures were comparable to those of PP.

In the second heating scan, the inner and outer layers retained similar thermal profiles, whereas the thermogram of the middle layer exhibited double melting endotherms at 154 °C and 163 °C. This behaviour is associated with different crystalline domains, including variations in crystal forms, crystal perfection, and phase reorganization. Similar findings have been reported in the literature, linking these phenomena to processing parameters, fabric production technology (melt-blowing or spun-bonding), and the molecular structure of PP, which influences the morphology of nonwoven fabrics [47,48].

The thermal stability of the masks was examined by TGA. Figure 1d depicts the weight-loss percentage as a function of temperature for the three layers of the disposable masks. The middle layer exhibited the typical degradation behaviour of pure PP, undergoing a single-step degradation that began at approximately 303 °C, with a maximum degradation temperature (ΔT_max_) of 472.8 °C.

Conversely, the inner and outer layers exhibited two distinct weight-loss events. The first degradation step, starting at approximately 280 °C with ΔT_max_ = 458.9 °C, was attributed to PP. The second weight-loss event, beginning at approximately 590 °C with ΔT_max_ = 662.2 °C, was associated with the decomposition of the CaCO_3_ filler, leaving a final residue of approximately 2.8 wt.% at 800 °C. This residue corresponded to a CaCO_3_ loading of approximately 5 wt.%.

These results were further corroborated by the density values of the three mask layers, which were measured as follows: 0.931 g/cm^3^ for the inner layer, 0.912 g/cm^3^ for the middle layer, and 0.930 g/cm^3^ for the outer layer.

### 3.2. Infrared Spectroscopic Analysis of PMB Mixtures

**Figure 2:**
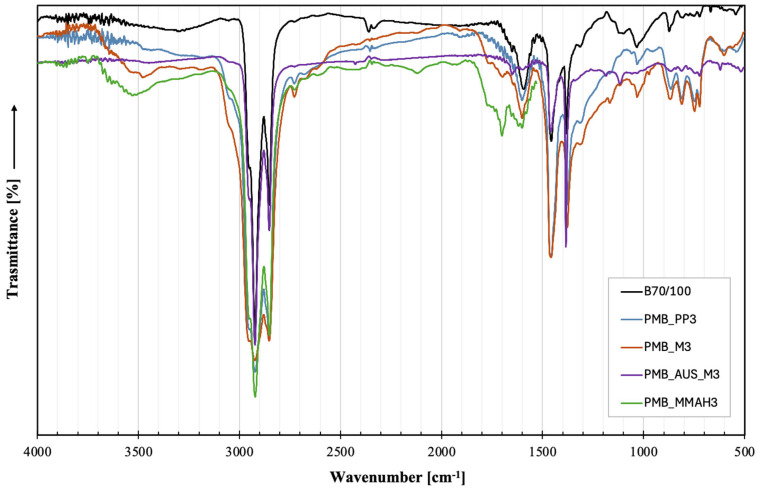
FT-IR spectra of pure B_70/100_ bitumen and all PMB mixtures with 3 wt.% polymeric modifiers.

The spectrograms of PMBs and their pure components are shown in Figures S2–S5 in the Supplementary Materials. To better highlight the differences resulting from the various PMB production methods, the spectrograms of bitumen containing 3 wt.% polymeric modifiers (PMB_PP3, PMB_M3, PMB_AUS_M3, PMB_MMAH3) were isolated and are presented in Figure 2. This percentage was chosen because, being among the highest, it was best suited to emphasize any differences in the spectrograms. Using 5 wt.% would lead to dominant viscosity and network effects, potentially masking these differences; therefore, 3 wt.% was selected as the most representative concentration for comparison.

All PMB mixtures exhibited the characteristic peaks of pure bitumen. 

Specifically, the most pronounced peaks were observed at 2924 cm^−1^ and 2855 cm^−1^ (attributed to CH stretching in the CH_3_ group), 1603 cm^−1^ (C = C stretching in aromatics), 1458 cm^−1^ (CH bending in CH_2_), and 1376 cm^−1^ (CH bending in CH_3_). The presence of shoulders at 1029 cm^−1^ was attributed to -CH_3_ substituents on aromatic rings, consistent with findings of Yoon et al. [49].

PMB mixtures containing MAH-functionalized shredded masks displayed additional absorption bands attributable to MAH, specifically at 3400–3200 cm^−1^ (carboxylic O-H stretching of dicarboxylic maleic acid) and 1770–1690 cm^−1^ (C=O stretching of the anhydride ring and dicarboxylic maleic acid, respectively) [50].

The absence of new peaks or peak shifts suggested that, in all modified bitumens, the interaction between the polymer phase and the bitumen was purely physical, as also reported by Nivitcha et al. [51], with no thermo-oxidative degradation occurring during the mixing phase. This finding is crucial, as oxidation of bitumen would lead to excessive embrittlement of the matrix, compromising its long-term stability. Furthermore, bitumens modified with high additive concentrations (3–5 wt.%) exhibited variations in the relative intensities of the 1458 cm^−1^ and 1376 cm^−1^ peaks, suggesting morphological changes related to the redistribution of aromatic compounds between the asphaltenic and polymeric phases.

### 3.3. PMBs Mixtures Characterization

#### 3.3.1. Viscosity

Pure bitumen and modified bituminous mixtures with different compositions were tested for viscosity, penetration strength, and softening point, which were chosen as performance indicators [34].

**Figure 3:**
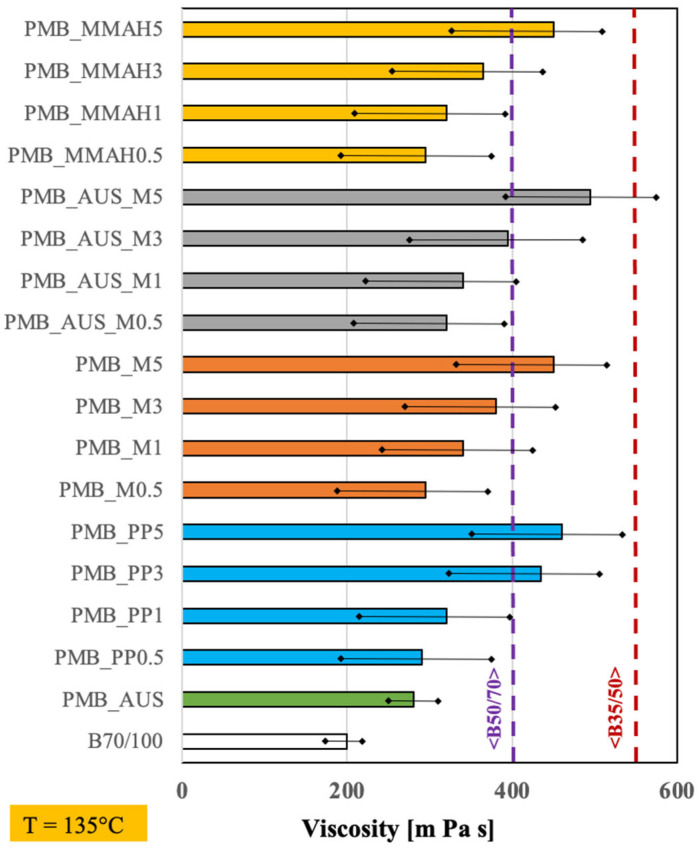
Average viscosity measurements at 135 °C.

The apparent viscosity values at 135 °C are shown in Figure 3.

As expected, all PMBs exhibited a higher apparent viscosity compared to pure bitumen. The viscosity increased by approximately 50% with a polymer phase loading of just 0.5 wt.%. It continued to rise progressively with increasing modifier content, with only slight differences due to the type of compatibilizer used. At 5 wt.%, all mixtures significantly exceeded the viscosity of the reference B_50/70_ bitumen, although none reached the viscosity levels of superior commercial products (e.g., B_35/50_). The highest viscosity improvement was observed in the PMB_AUS_M5 sample, which contained 5 wt.% shredded masks and the AUS compatibilizer.

#### 3.3.2. Penetration Force

**Figure 4:**
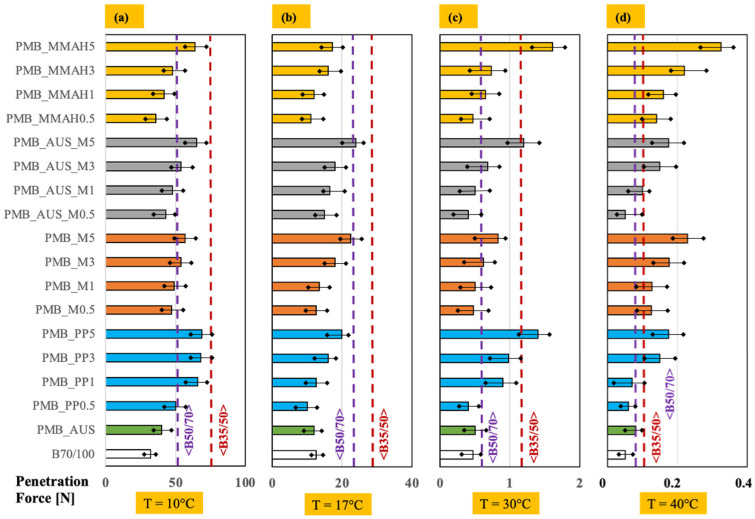
Average penetration force (N) for PMB mixtures at (**a**) 10, (**b**) 20, (**c**) 30 and (**d**) 40 °C.

As described in Section 2.4, penetration testing provides information on bitumen consistency at a given temperature. Following the modification of bitumen with a polymer matrix, the objective was to decrease the penetration depth (i.e., increase penetration force). The results are presented in Figure 4.

At room temperature (20 °C, Figure 4b), all mixtures exhibited higher penetration force values than the reference B_70/100_ bitumen. Specifically, the PMB_PP samples exceeded the penetration force of B_70/100_ in three out of four cases, while PMB_M and PMB_AUS_M exceeded it in all cases. PMB_MMAH also outperformed B_70/100_ in three out of four cases. Conversely, PMB_AUS exhibited a penetration force slightly below that of B_70/100_.

The beneficial effect of increasing the polymer matrix percentage was evident, with penetration force consistently rising from 0.5 wt.% to 5 wt.%. Notably, PMB_AUS_M5 and PMB_M5 at 5 wt.% reached penetration force values comparable to the commercial B_50/70_ bitumen.

The effect of temperature was also investigated (Figure 4a,c,d). As expected, penetration force increased at lower temperature (10 °C) due to the increased rigidity of bitumen, while it decreased at higher temperatures (30 °C and 40 °C) as the material became more fluid.

At 10 °C (Figure 4a), all mixtures exhibited higher penetration force than B_70/100_, and many even outperformed B_50/70_. Additionally, five mixtures (PMB_PP5, PMB_PP3, PMB_PP1, PMB_AUS_M5, PMB_MMAH5) approached the penetration force of the commercial B_35/50_ category. At 30 °C (Figure 4c), almost all PMBs maintained higher penetration force than B_70/100_, with some exceeding the values of B_50/70_ and B_35/50_ (e.g., PMB_PP5, PMB_AUS_M5, and PMB_MMAH5). Similar trends were observed at 40 °C (Figure 4d). These findings align with literature studies linking improved penetration resistance to the formation of a polymer network, which enhances mechanical stability at elevated temperatures [52,53].

The superior performance of PMB_MMAH at temperatures above 20 °C can be attributed to a reduction in molecular mobility. This effect is more pronounced at higher temperatures due to network interactions between bitumen components, promoted by MAH and its dicarboxylic maleic acid (from ring opening), via hydrogen bonding and π-π interactions with bitumen species [54,55].

**Figure 5:**
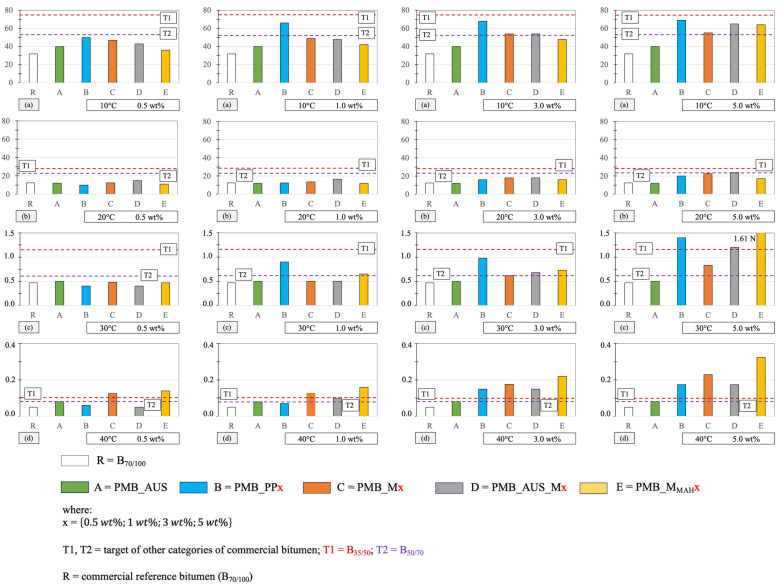
Average penetration force values (N) for PMB mixtures as temperature, polymer typology added to the mixture and percentage (0.5 wt.%, 1 wt.%, 3 wt.%, 5 wt.%) changes. Penetration force values at (**a**) 20 °C (room temperature), (**b**) 10 °C; (**c**) 30 °C; (**d**) 40 °C.

Figure 5 further illustrates penetration force trends at various temperatures and polymer matrix percentages.

At 10 °C (Figure 5a), among the mixtures with a lower polymer phase content (0.5 wt.%; 1 wt.%), those with the highest penetration force values were PMB_PP. PMB_AUS also exceeded the reference value of B_70/100_. At higher polymer contents (3 wt.%; 5 wt.%), an increase in penetration force was also observed for the other PMB mixtures (PMB_M, PMB_AUS_M, PMB_MMAH), with the maximum increase recorded at 5 wt.%. All modified PMB mixtures performed better than the reference B_70/100_.

At an ambient temperature of 20 °C (Figure 5b), penetration force values were generally comparable to those of the reference B_70/100_. However, at 5 wt.% polymer content, the penetration force showed a more significant increase compared to B_70/100_.

At 30 °C (Figure 5c), the PMB_PP and PMB_MMAH mixtures exhibited the highest penetration force values, significantly exceeding that of the reference B_70/100_. Similar trends were observed at 40 °C (Figure 5d). These findings highlight that maleic anhydride-based PMB mixtures performed better at high temperatures while still maintaining superior performance compared to B_70/100_ at 10 °C and 20 °C.

#### 3.3.3. Softening Measurements

**Figure 6:**
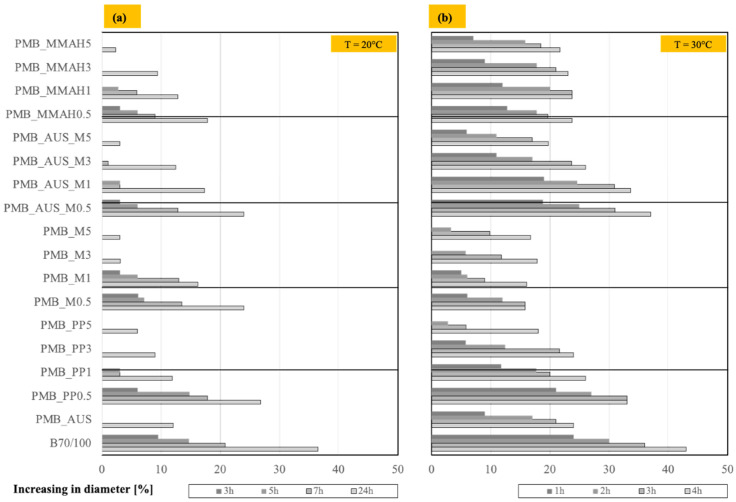
Average softening measurements in terms of increasing Diameter Percentage (%) for all the realized PMB mixtures at 20 °C (**a**) and 30 °C (**b**), and by varying the hours (standard deviations < 5%).

Softening tests were conducted to determine sample diameter expansion at different temperatures. A smaller diameter increase indicates higher resistance to softening. The results at 20 °C are shown in Figure 6a.

All PMB mixtures exhibited smaller diameter increases than B_70/100_. Mixtures containing 3 wt.% and 5 wt.% additives (except for the one with 3 wt.% compatibilized masks) showed no diameter change for up to 24 h. Additionally, PMBs with 3 wt.% and 5 wt.% additives outperformed the three pure bitumens.

Among the PMBs with 5 wt.% additives, those containing shredded masks exhibited lower diameter increases than those with 5 wt.% PP. At high additive percentages (3 wt.% and 5 wt.%), the unmodified shredded mask PMBs demonstrated the best results.

At 30 °C (Figure 6b), the diameter increases stabilized after 4 h of testing. PMBs with B70/100 bitumen and unmodified masks (PMB_M) outperformed other PMB mixtures at 0.5 wt.–3 wt.%, while at 5 wt.%, PMB_M5 was slightly less effective than PMB_PP5.

At 40 °C, diameter measurements remained unchanged after 48 h, suggesting high thermal resistance.

### 3.4. Overall Performance Evaluation

The results presented in this section are interpreted from a global perspective, following the methodology described in Section 2.5.

**Figure 7:**
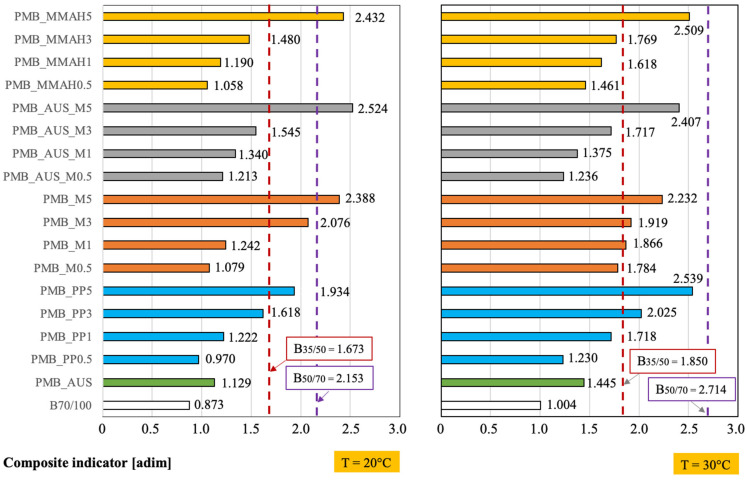
Comparison of the overall performance of PMB mixtures with the reference B_70/100_ bitumen and other commercial target bitumens (B_35/50_, B_50/70_) at room temperature (20 °C) and at 30 °C. The benefit associated with the composite indicator (CI) is greater the higher its numerical value.

At room temperature (20 °C, Figure 7), all PMB mixtures exhibited higher CI values than the reference B_70/100_ bitumen.

Overall, the best-performing mixtures were PMB_M, PMB_AUS and PMB_MMAH. The maleic anhydride-based mixtures demonstrated outstanding performance, even surpassing the highest commercial bituminous targets (B_50/70_; B_35/50_).

At 30 °C (Figure 7), the best overall performance was observed for the PMB_PP5 mixture (containing 5 wt.% polymeric solids), although only slightly higher than the other mixtures, particularly the maleic anhydride-based PMB_MMAH5. All mixtures containing 5 wt.% polymeric solids (PMB_MMAH5; PMB_AUS_M5; PMB_AUS) exhibited higher CI values than both B_70/100_ and B_50/70_.

Furthermore, Table 3 presents a comparison between one of the best-performing PMB mixtures (PMB_MMAH5) and conventional PP-based blends.

This comparison revealed that the PMB_MMAH5 mixture simultaneously meets all the performance criteria of the mixture containing 3 wt.% virgin PP. These findings suggest that MAH-grafted shredded masks can effectively replace virgin PP polymers typically used for bitumen modification, given the strong performance of PMB_MMAH systems at both 20 °C and 30 °C.

## 4. Conclusions

The results obtained in this study demonstrate that polypropylene recovered from unused surgical masks can be effectively integrated into bitumen through appropriate compatibilization strategies. Among the formulations investigated, the maleic anhydride-functionalized mask-derived PP (PMB_MMAH) provided the most significant performance enhancement, showing an increase in the softening point of approximately 10–15 °C and a viscosity rise of about 20–30% in the 10–40 °C range, together with a reduction in penetration, indicating greater stiffness and resistance to deformation compared to the base binder (B_70/100_). These findings indicate that mask-derived PP can represent a technically viable and more sustainable alternative to virgin PP in polymer-modified bitumen production, contributing simultaneously to waste reduction and improved binder performance.

## 5. Limitations and Future Research Directions

While PMB_MMAH showed the most favourable performance, the long-term ageing behaviour has yet to be assessed. Ageing simulations such as Rolling Thin-Film Oven Test (RTFOT) and Pressure Ageing Vessel (PAV), together with rheological analyses including Dynamic Shear Rheometer (DSR) and Multiple Stress Creep Recovery (MSCR) testing, will be required to evaluate performance retention and storage stability under service conditions. The Composite Indicator was used solely for internal comparison; different weighting schemes may be applied in application-specific evaluations. The calcium carbonate (CaCO_3_) filler inherently present in the mask-derived polypropylene was retained, and its contribution is already reflected in the comparative performance trends reported.

This study employed unused surplus surgical masks, so issues related to contamination or sanitization of post-consumer materials fall outside its scope.

Future work will also include LCA to quantify the environmental benefits of replacing virgin PP with surplus mask-derived PP.

## Figures and Tables

**Table 1:** General properties of the base bitumens determined in accordance with EN 1426 (penetration) [36], EN 1427 (softening point) [37] and EN 12591 [38].

Property	Unit	Bitumen		
		B_35/50_	B_50/70_	B_70/100_
Penetration at 25 °C	1/10 mm	35–50	50–70	70–100
Softening Point (Ball and Ring)	°C	50–58	46–54	43–51
Penetration Index	-	−1.5–0.7	−1.5–0.7	−1.5–0.7
Specific gravity 25 °C/25 °C	-	1.00–1.10	1.00–1.10	1.00–1.07
Flash Point COC	°C	250	250	250
Dynamic Viscosity at 60 °C	Pa·s	225	145	90
Dynamic Viscosity at 135 °C	mPa·s	370	295	230
Solubility in organic solvents	wt.%	99.0	99.0	99.0

**Table 2:** Nomenclature and composition of PMBs mixtures.

Sample Name	Composition (wt.%)				
	B_70/100_	Polymer Modifier			
		AUS	PP	M	MMAH ^(a)^
PMB_AUS	95.0	5.0		-	-
PMB_PP0.5	99.5	-	0.5	-	-
PMB_PP1	99.0	-	1.0	-	-
PMB_PP3	97.0	-	3.0	-	-
PMB_PP5	95.0	-	5.0		-
PMB_M0.5	99.5	-	-	0.5	-
PMB_M1	99.0	-	-	1.0	-
PMB_M3	97.0	-	-	3.0	-
PMB_M5	95.0	-	-	5.0	-
PMB_AUS_M0.5	99.5	5	-	0.5	-
PMB_AUS_M1	99.0	5	-	1.0	-
PMB_AUS_M3	97.0	5	-	3.0	-
PMB_AUS_M5	95.0	5	-	5.0	-
PMB_MMAH0.5	99.5	-	-	-	0.5
PMB_MMAH1	99.0	-	-	-	1.0
PMB_MMAH3	97.0	-	-	-	3.0
PMB_MMAH5	95.0	-	-	-	5.0

^a^ Three-layer surgical mask grafted with 0.8 wt.% of MAH.

**Table 3:** Performance comparison of PMB_MMAH5 with conventional PP blends.

Parameter		Degree to Which the PMB_MMAH5 Mixture Reaches the Performance Values of Conventional PP-Based Mixtures		
		PMB_PP1	PMB_PP3	PMB_M_MAH5_
Viscosity		Completely	Completely	Completely
Penetration	10 °C	Completely	Completely	Completely
	20 °C	Completely	Completely	Completely
	30 °C	Completely	Completely	Completely
	40 °C	Completely	Completely	Completely
Softening	10 °C	Completely	Completely	Completely
	30 °C	Completely	Completely	Partly

## Data Availability

The original contributions presented in this study are included in the article/Supplementary Material. Further inquiries can be directed to the corresponding author.

## Supplementary Materials

The following supporting information can be downloaded at: https://www.mdpi.com/article/10.3390/polym17233110/s1. Figure S1: Mask for conducting softening tests, made with a series of concentric rings, with a diameter of 4 cm, an external diameter of 10 cm and a radius increase of 2 mm; Figure S2: FT-IR spectrograms of B_70/100_ and PP mixtures (PMB_PP); Figure S3: FT-IR spectrograms of B_70/100_ and masks (PMB_M); Figure S4: FT-IR spectrograms of B_70/100_ and compatibilized masks (PMB_AUS_M); Figure S5: FT-IR spectrograms of B_70/100_ and maleized masks (PMB_MMAH).

## Abbreviations

The following abbreviations are used in this manuscript:

ATR/FT-IR	Attenuated total reflectance infrared spectroscopic analysis
CI	Composite indicator
DCP	Dicumyl peroxide
DSC	Differential scanning calorimetry
EBA	Ethylene–butyl acrylate
EVA	Ethylene–vinyl acetate
AUS	Intune
KOH	Potassium hydroxide
HCl	Hydrochloric acid
MAH	Maleic anhydride
PE	Polyethylene
PMB	Polymer-modified bitumen
PP	Polypropylene
RLLDPE	Recycled linear low-density polyethylene
SBS	Styrene–butadiene–styrene
SEBS	Styrene–ethylene–vinyl acetate
SIS	Styrene–isoprene–styrene
TGA	Thermogravimetric analysis
Tg	Glass transition temperature
Tm	Melting temperature
