# Supplementary material for: Valorization of Unused, Expired Surgical Masks in Polymer-Modified Bitumens Using Maleic Anhydride as a Compatibilizer
Source: Polymers (Basel). 2025 Nov 23;17(23):3110. doi: 10.3390/polym17233110 (PMC12693888; doi:10.3390/polym17233110)
Supplement: Supplementary file 1 [file polymers-17-03110-s001.zip › polymers-3899075-supplementary.pdf]

SUPPLEMENTARY MATERIAL

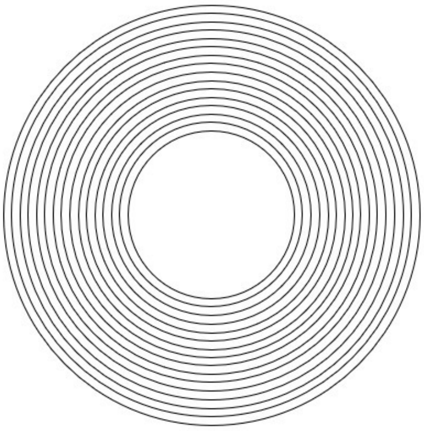

Figure S1. Mask for conducting softening tests, made with a series of concentric rings, with a diameter of 4 cm, an external diameter of 10 cm and a radius increase of 2 mm.

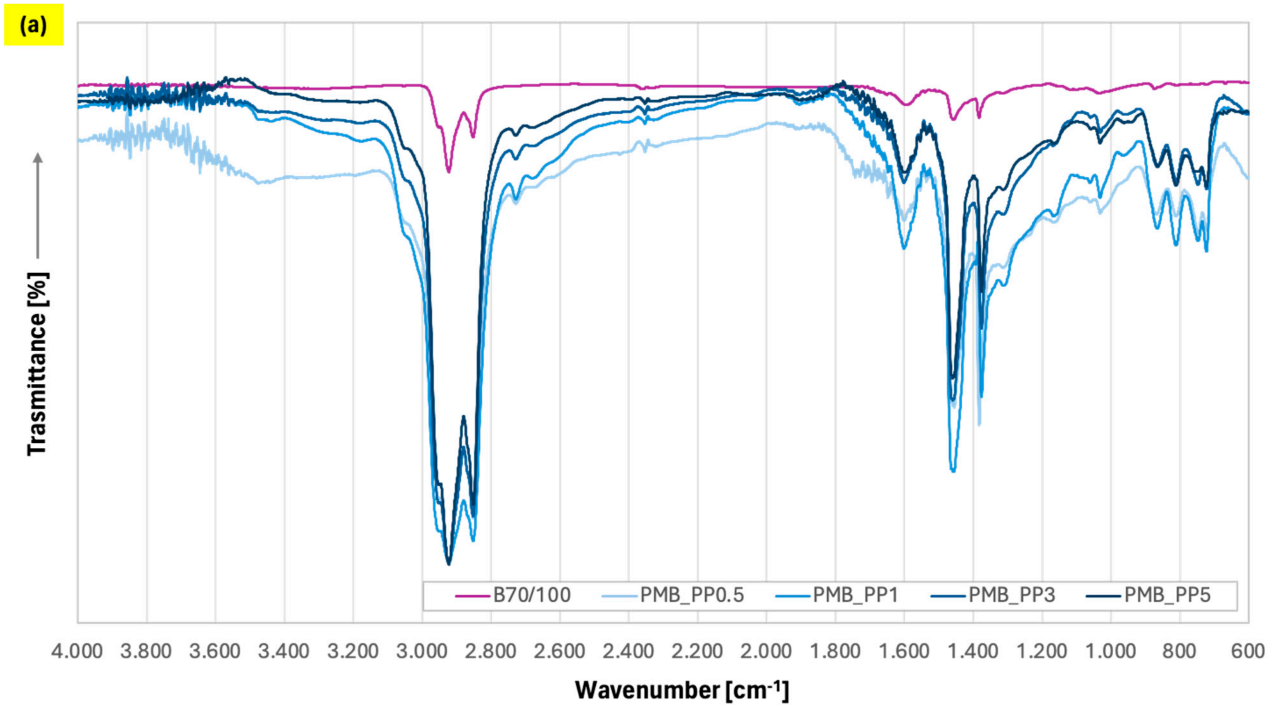

Figure S2. FT-IR spectrograms of $B_{70/100}$ and PP mixtures (PMB_PP).

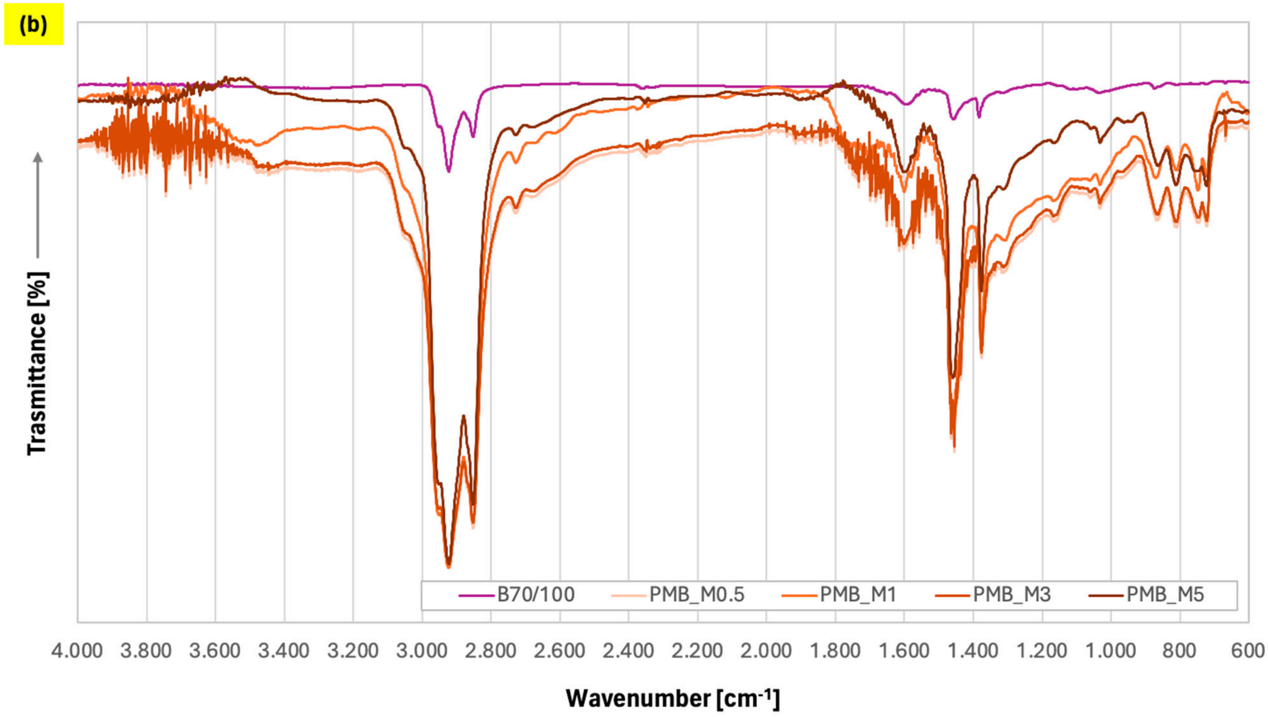

Figure S3. FT-IR spectrograms of $B_{70/100}$ and masks (PMB_M).

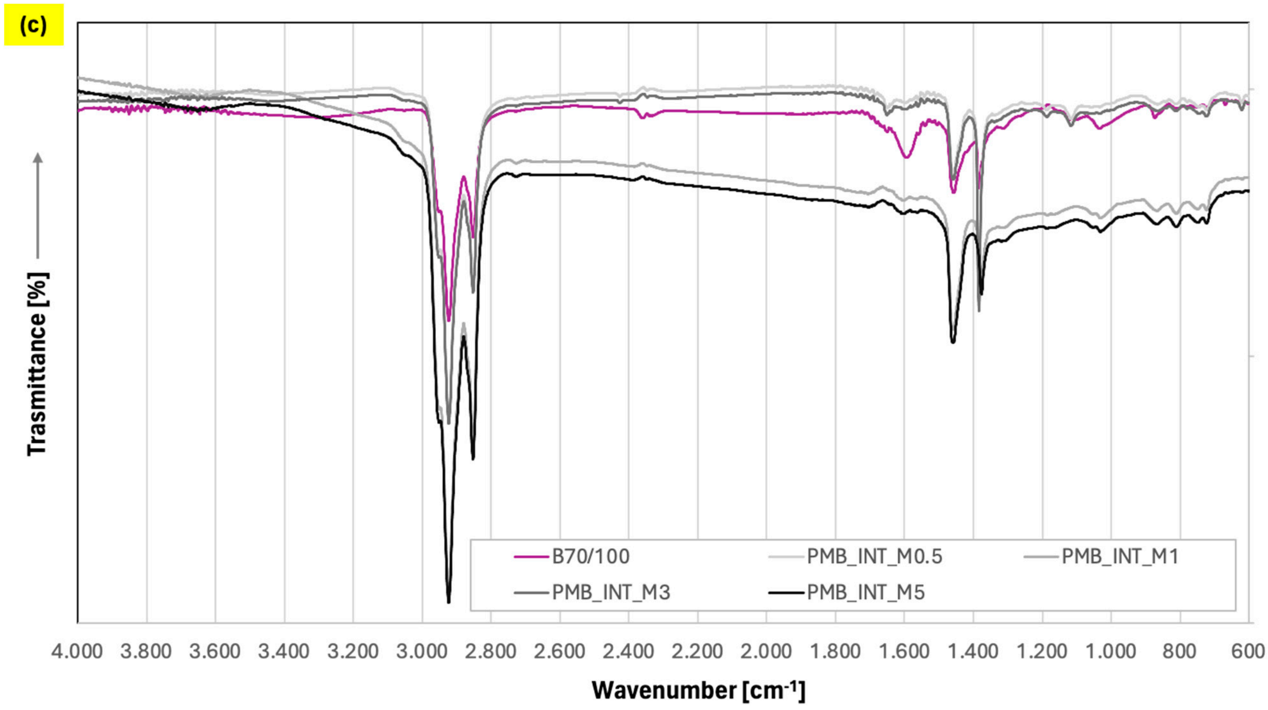

Figure S4. FT-IR spectrograms of $B_{70/100}$ and compatibilized masks (PMB_AUS_M).

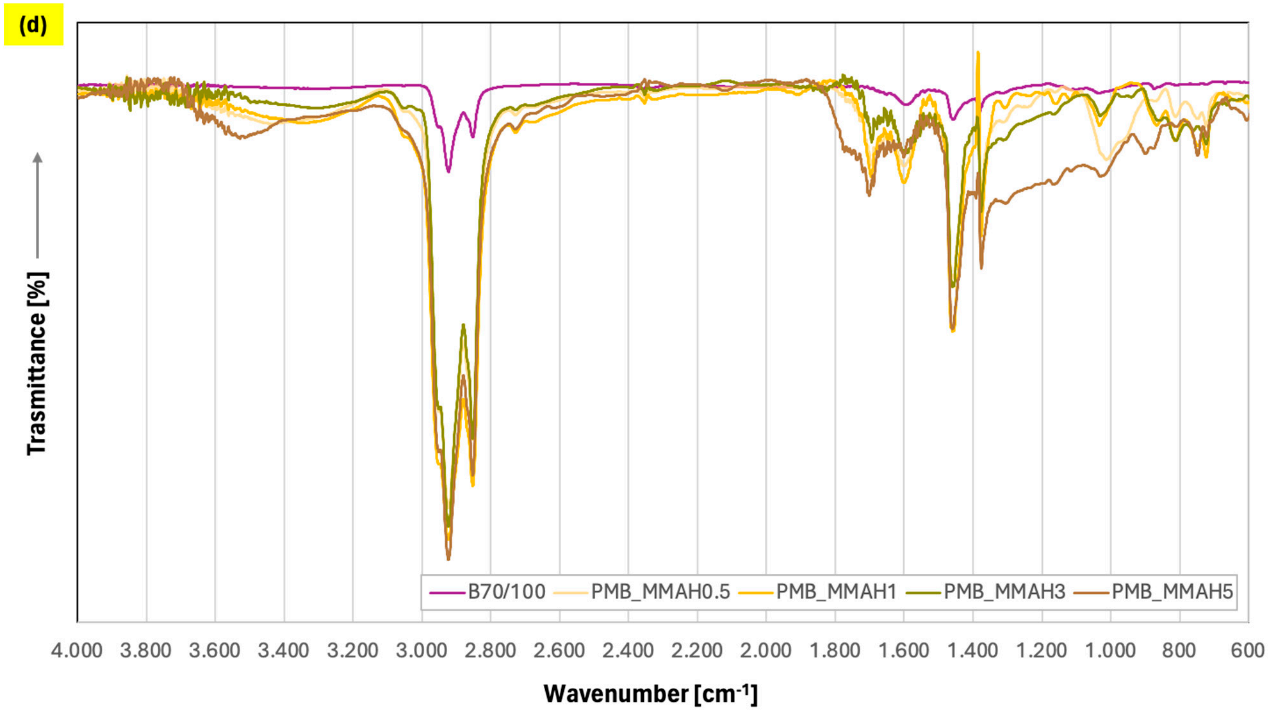

Figure S5. FT-IR spectrograms of $B_{70/100}$ and maleized masks (PMB_MMAH).
